# Supplementary material for: Location- and feature-based selection histories make independent, qualitatively distinct contributions to urgent visuomotor performance
Source: bioRxiv. 2024 May 30:2024.05.29.596532. Preprint. [Version 1] doi: 10.1101/2024.05.29.596532 (PMC11160778; doi:10.1101/2024.05.29.596532)
Supplement: Supplementary file 1 [file NIHPP2024.05.29.596532v1-supplement-1.pdf]

bioRxiv preprint doi: https://doi.org/10.1101/2024.05.29.596532; this version posted May 31, 2024. The copyright holder for this preprint (which was not certified by peer review) is the author/funder, who has granted bioRxiv a license to display the preprint in perpetuity. It is made available under aCC-BY 4.0 International license.

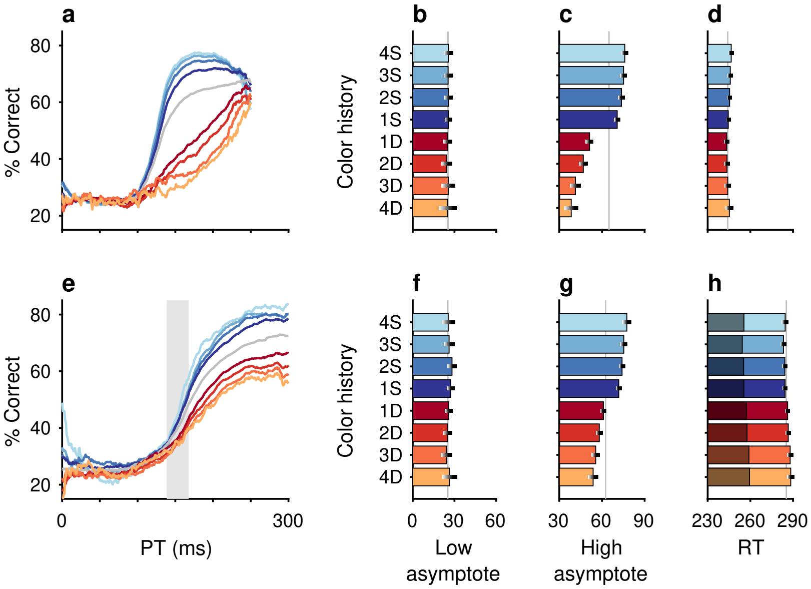

Figure 1 — figure supplement 1. Target color repetition affects perceptual accuracy in a consistent manner across subjects. (**a**–**d**) Task performance for monkey C. (**e**–**h**) Task performance for monkey N. (**a**, **e**) Individual subject tachometric curves denoting performance on the current trial conditioned on histories of preceding trials having the same target color (S, blue spectrum; 1–4 preceding trials) or different target color (D, red spectrum; 1–4 preceding trials). Average performance is shown in gray. The vertical gray shade represents the −29 ms shift used to align the data from monkey N with those from monkey C; it represents the centerpoints of the average tachometric curve of monkey N before (right edge of the shade) and after the shift (left edge of the shade). (**b**, **f**) Choice accuracies in short-PT trials (PT < 100 ms, low asymptote). Error bars represent 95% CIs. Gray vertical lines denote values for the average tachometric curves. (**c**, **g**) Choice accuracies in long-PT trials (PT > 150 ms, high asymptote). Error bars represent 95% CIs. Gray vertical lines denote values for the average tachometric curves. (**d**, **h**) Average RT ± 1 SEM for each history condition. The gray vertical lines denote the average RTs across all trials included in the analysis. Dark bars in **h** depict the aligned RT of monkey N shifted by −29 ms.

947

bioRxiv preprint doi: https://doi.org/10.1101/2024.05.29.596532; this version posted May 31, 2024. The copyright holder for this preprint (which was not certified by peer review) is the author/funder, who has granted bioRxiv a license to display the preprint in perpetuity. It is made available under aCC-BY 4.0 International license.

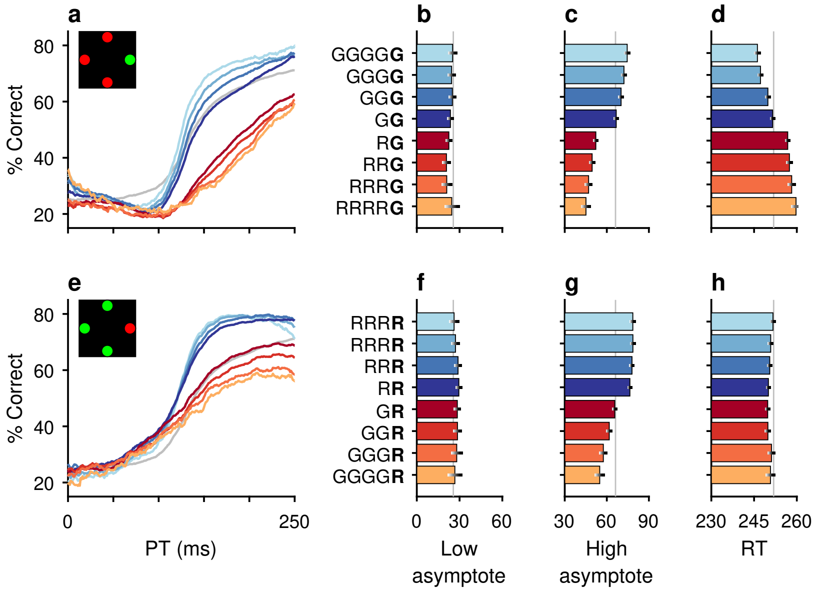

Figure 1 — figure supplement 2. Target color repetition affects decision accuracy in a consistent manner across colors. (**a**) Tachometric curves denoting performance on green-target trials (see target array inset) conditioned on histories of preceding trials having the same target color (blue spectrum; 1–4 preceding green trials) or a different target color (red spectrum; 1–4 preceding red trials). Average performance is shown in gray. (**b**) Choice accuracy in short-PT trials (PT $<$ 100 ms, low asymptote) conditioned on color history. Error bars represent 95% CIs across trials. Gray vertical line denotes value for the average tachometric curve. (**c**) Choice accuracy in long-PT trials (PT $>$ 150 ms, high asymptote) conditioned on color history. Error bars represent 95% CIs across trials. Gray vertical line denotes value for the average tachometric curve. (**d**) Average RT $\pm$ 1 SEM conditioned on color history. Gray vertical line denotes average RT across all trials included in the analysis. (**e**–**h**) As in **a**–**d** but for red targets among green distracters (see target array inset).

948

bioRxiv preprint doi: https://doi.org/10.1101/2024.05.29.596532; this version posted May 31, 2024. The copyright holder for this preprint (which was not certified by peer review) is the author/funder, who has granted bioRxiv a license to display the preprint in perpetuity. It is made available under aCC-BY 4.0 International license.

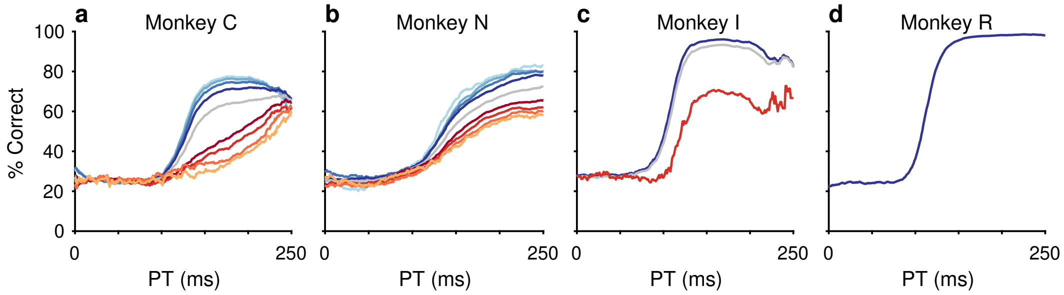

Figure 1 — figure supplement 3. Variability in performance across animals and rule sets. (**a**, **b**) Task performance for monkey C (**a**) and monkey N (**b**) in the current study; during the CO task, target colors and locations were randomly sampled across trials. Tachometric curves show performance on the current trial conditioned on the histories of preceding trials having the same target color (S, blue spectrum; 1S–4S sequences; dark to light) or a different target color (D, red spectrum; 1D–4D sequences; dark to light). (**c**) Task performance for monkey I from a previous study (Scerra et al., 2019). This subject ran a version of the CO task in which target locations were randomly sampled but target color remained constant for each block of (10–200) trials. The red curve represents average performance from the first 5 trials following a color switch. The blue curve denotes average performance on a block of trials but excluding the first 5 trials after a color switch. (**d**) Task performance for monkey R from a previous study (Scerra et al., 2019). This subject ran a version of the CO task in which target locations were randomly sampled but target color did not change. Bin width is 50 ms for all curves. The gray trace in each panel shows the average tachometric curve obtained from all the trials considered.

949
